# Computational design and evaluation of the mechanical and electrical behavior of a piezoelectric scaffold: a preclinical study

**DOI:** 10.3389/fbioe.2023.1261108

**Published:** 2024-01-11

**Authors:** Vahid Badali, Sara Checa, Manfred M. Zehn, Dragan Marinkovic, Melika Mohammadkhah

**Affiliations:** ^1^ Department of Structural Mechanics and Analysis, Technische Universität Berlin, Berlin, Germany; ^2^ Julius Wolff Institute, Berlin Institute of Health, Charité—Universitätsmedizin Berlin, Berlin, Germany

**Keywords:** large bone defect, piezoelectric scaffold, bone regeneration, finite element analysis (FEA), scaffold design

## Abstract

Piezoelectric scaffolds have been recently developed to explore their potential to enhance the bone regeneration process using the concept of piezoelectricity, which also inherently occurs in bone. In addition to providing mechanical support during bone healing, with a suitable design, they are supposed to produce electrical signals that ought to favor the cell responses. In this study, using finite element analysis (FEA), a piezoelectric scaffold was designed with the aim of providing favorable ranges of mechanical and electrical signals when implanted in a large bone defect in a large animal model, so that it could inform future pre-clinical studies. A parametric analysis was then performed to evaluate the effect of the scaffold design parameters with regard to the piezoelectric behavior of the scaffold. The designed scaffold consisted of a porous strut-like structure with piezoelectric patches covering its free surfaces within the scaffold pores. The results showed that titanium or PCL for the scaffold and barium titanate (BT) for the piezoelectric patches are a promising material combination to generate favorable ranges of voltage, as reported in experimental studies. Furthermore, the analysis of variance showed the thickness of the piezoelectric patches to be the most influential geometrical parameter on the generation of electrical signals in the scaffold. This study shows the potential of computer tools for the optimization of scaffold designs and suggests that patches of piezoelectric material, attached to the scaffold surfaces, can deliver favorable ranges of electrical stimuli to the cells that might promote bone regeneration.

## 1 Introduction

Bone defects over a specific size do not heal on their own and result in non-unions (Geris et al., 2010; Nicholson et al., 2021; Wildemann et al., 2021). Large bone defects still remain a clinical challenge, and the currently used treatment procedures, including autologous bone grafting, exhibit several drawbacks such as the lack of sufficient material or donor side morbidity (Zimmermann and Moghaddam, 2011; García-Gareta et al., 2015; Sanz-Sánchez et al., 2022). To enhance bone regeneration and overcome the drawbacks of other treatment strategies, various bone tissue scaffolds, which differ in design and employed materials, have been developed over the last decades as appealing alternatives due to their versatility in the design process and potential to customize to the patient-specific defect situation (Akkouch et al., 2011; Liu et al., 2013; Zadpoor, 2015; Huang et al., 2019; Shick et al., 2019; Chen et al., 2020; Milovanović et al., 2020; Deng et al., 2021). Several studies have investigated the influence of scaffold mechanical properties on the biology of the regeneration process and have shown their potential to support bone defect healing (e.g., Pobloth et al., 2018; Reznikov et al., 2019; Tan et al., 2023). Among them, piezoelectric scaffolds have shown a great potential to support bone regeneration (Damaraju et al., 2017; Tandon et al., 2018).

In piezoelectric materials, a mechanical deformation causes the formation of a net dipole moment and subsequently polarization of the material, which is known as direct piezoelectric effect. Piezoelectric materials have extensively been investigated for various industrial applications such as microphones, hydrophones, sensors and actuators (Topolov et al., 2015; Noll et al., 2019) as well as health monitoring, drug delivery, and biomedical devices (Liu et al., 2018; Qing et al., 2019; Bußmann et al., 2021). Biological tissues such as bone, muscle and tendon also present piezoelectric properties (Stapleton et al., 2016; Lay et al., 2021). The first attempt to use piezoelectric materials for bone implants was made in the 1980s (Park et al., 1981). Piezoelectric materials have experimentally shown to be capable of altering cellular behavior through surface charges generated in response to deformation (physiological movements) (Ribeiro et al., 2017). Electromechanical effects in bone and their role in modulating cellular behavior and tissue remodeling processes have been also widely investigated (Qu et al., 2006; Fernández et al., 2012; Garzón-Alvarado et al., 2012; Cerrolaza et al., 2017). In addition, the effects of electrical stimulation on bone healing have broadly been evaluated both *in vitro* and *in vivo*, which have shown that electrical stimuli can promote and stimulate osteogenic activity (Ciombor and Aaron, 2005; Khalifeh et al., 2018; Leppik et al., 2018; Hu et al., 2019; Li et al., 2020). Several experimental studies have clearly shown the benefits of piezoelectric scaffolds for tissue regeneration (Rajabi et al., 2015; Azimi et al., 2020; More et al., 2020; Zaszczynska et al., 2020; Goonoo and Bhaw-Luximon, 2022), particularly for bone healing (Damaraju et al., 2017; Qi et al., 2021; Zhao et al., 2020; Tariverdian et al., 2019; Liu et al., 2020b; D’Alessandro et al., 2021), however the mechanisms behind this enhanced bone healing response remains poorly understood.

Scaffold design is a challenging task since a large number of parameters play a role on the healing outcome; such as scaffold stiffness (Breuls et al., 2008), porosity and pore shape (Razi et al., 2012; Zhang et al., 2014; Zhang et al., 2016). Different piezoelectric scaffold designs have been tested in pre-clinical studies for their potential to enhance bone regeneration mainly using experimental trial and error approaches (Liu et al., 2020b; Marques-Almeida et al., 2020; Polley et al., 2020), which are expensive, time-consuming and ethically questionable. Analysis of the scaffold performance using computer models has on the other hand the potential to support the design of these structures and reduce the number of *in vivo* experiments. In addition, it will be a prerequisite for the design of personalized scaffolds enabling bone defect healing.

Several computer models have been developed to support the design of scaffolds for bone regeneration (Podshivalov et al., 2014; Carlier et al., 2015; Arjunan et al., 2020; Metz et al., 2020; Jaber et al., 2022; Perier-Metz et al., 2022), however only one investigation has been performed to computationally model piezoelectric scaffold behavior (Jiang et al., 2022). Jiang et al., computationally modelled a scaffold completely made of piezoelectric material which resulted in voltages in the scale of thousands volts, which has been reported not to be suitable for bone regeneration (Bounds et al., 2018). Therefore, the aim of this study was 1) to design a piezoelectric scaffold that would provide favorable mechanical and electrical signals for the regeneration of bone, and 2) to identify key design features of piezoelectric scaffolds to provide optimal voltage ranges suitable for the bone regeneration process.

## 2 Materials and methods

**FIGURE 1:**
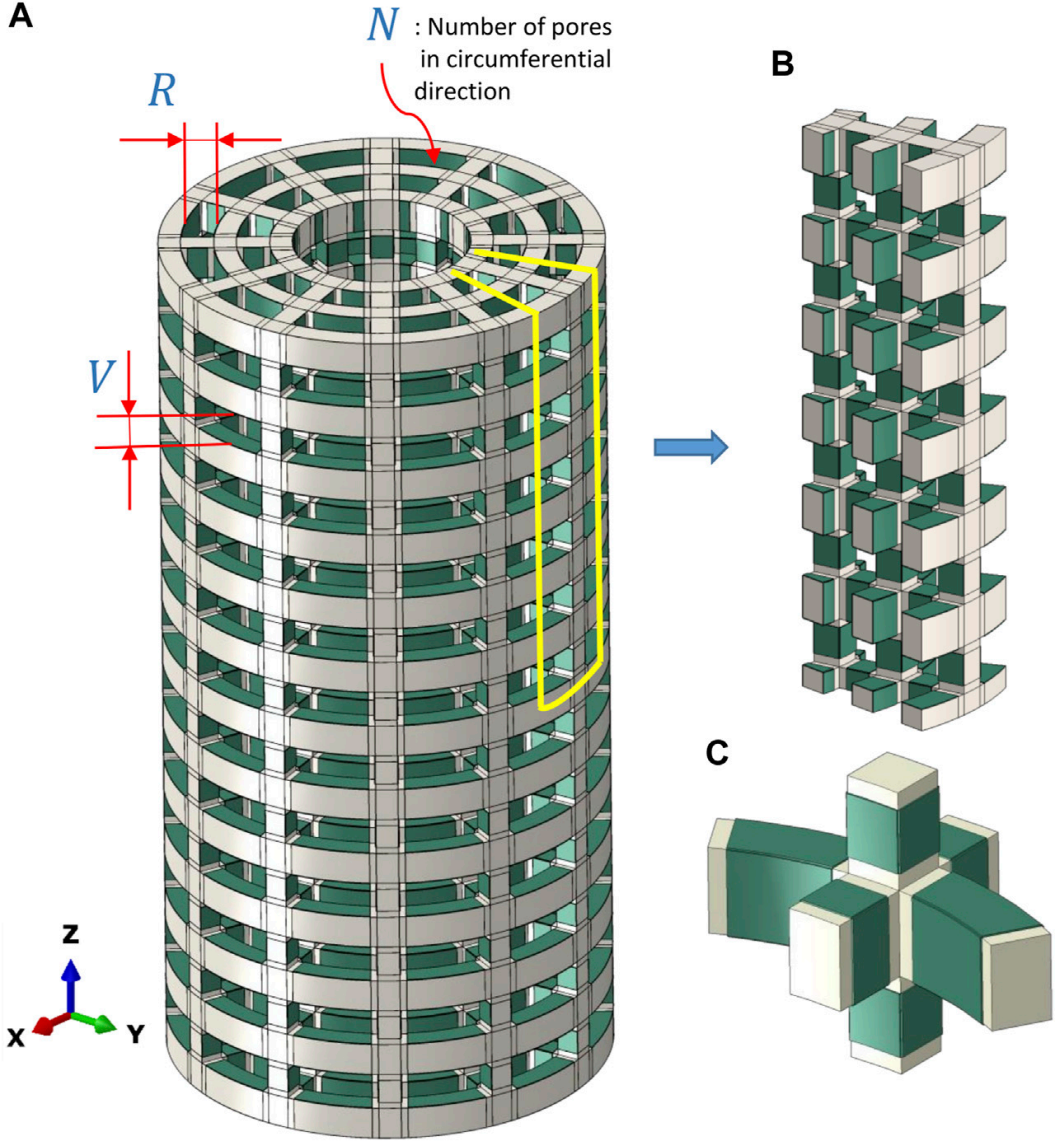
**(A)** Full model of the scaffold with bonded piezoelectric patches (green) and the defined geometrical parameters used in the present sensitivity study. **(B)** A portion (1/2*N*) of the model of the piezoelectric scaffold simulated due to symmetric conditions. **(C)** A closer view of piezoelectric patches (green) that were bonded to the scaffold surfaces facing the inner pores.

A scaffold was designed to fit into a large bone defect in a sheep tibia model used in a previously published experimental investigation (Pobloth et al., 2018). To fit into the defect area, the scaffold was designed with a cylindrical shape of 4 cm height and 2 cm diameter. A finite element strut-like scaffold model was developed using Abaqus/CAE 2020 (Dassault Systems Simulia Corp., RI, United States), which consisted of a non-piezoelectric material and piezoelectric patches bonded to the scaffold surfaces facing the inner pores (Figure 1). A range of scaffold designs were then tested to identify potential scaffold configurations that could lead to favorable mechanical and electrical signals.

### 2.1 Model geometry

Only the scaffold, without any material within the pores, was modelled using the finite element analysis. The designed scaffolds had a strut-like configuration with pore dimensions defined by the vertical (*V*) and radial sizes of the pores (*R*) (Figure 1A). In addition, the pore size was defined by the number of pores in the circumferential direction (*N*) (Figure 1A). Due to symmetry, only 1/*N* of the cross-section and 1/2 of the height was simulated (Figure 1B). Piezoelectric patches were simulated to be bonded to the scaffold surfaces facing the inner pores (Figure 1C).

### 2.2 Material properties

Material properties of titanium and polycaprolactone (PCL) used to model the scaffold, and barium titanate (BT) and Polyvinylidene fluoride (PVDF) used for the piezoelectric patches, are shown in Table 1. The piezoelectric charge constant *d*_ij_ expresses the amount of induced charge in the material in response to the stress applied (Table 1). The subscript i denotes the three directions of the polarization, while the subscript j refers to the applied stress (1, 2 and 3 denote normal stresses, while 4, 5 and 6 denote the corresponding shear stresses).

**TABLE 1:** Mechanical and piezoelectric properties of the materials used for the scaffold and the patches (Nix and Ward, 1986; Eshraghi and Das, 2010; Narita and Shindo, 2015; Perier-Metz et al., 2020).

	Young’s modulus (GPa)	Poisson’s ratio	*d*_31_ (pC/N)	*d*_32_ (pC/N)	*d*_33_ (pC/N)	*d*_15_ (pC/N)	*d*_24_ (pC/N)	Relative permittivity
Titanium	104	0.3	—	—	—	—	—	—
PCL	0.3	0.3	—	—	—	—	—	—
PVDF	2.7	0.35	21	1.5	−32.5	−27	−23	8.5
Barium titanate	112	0.3	−60	−60	140	260	260	1,450

The poling direction of the piezoelectric patches was considered to be through the thickness of each patch. The electric potential of the surface of the patches which are in contact with the scaffold was set to be zero.

### 2.3 Loading and boundary conditions

Loading and boundary conditions were derived from a previously developed finite element model of the experimental large bone defect stabilized with a plate (Perier-Metz et al., 2020). This model included the tibia (represented as a hollow cylinder), the gap and the external fixator. The designed scaffolds investigated in this study were virtually inserted in the gap area of the already existing finite element model to calculate the load share between the fixator and the scaffold (Supplementary Figure S1).

Physiological loading conditions (Perier-Metz et al., 2020) were applied to both bone ends (*z* direction), and load shares ranging from 110 N to 1100 N were determined for the PCL and titanium scaffolds (Supplementary Table S1). These loads were then applied on the top surface of the scaffold as a pressure load in the finite element model of the designed scaffolds. Symmetric boundary conditions were applied to the surfaces of (1/2N) of the model which were separated from the full model due to symmetry (Figure 1B). The scaffold apparent young modulus is determined by obtaining scaffold deflection in response to the applied pressure.

### 2.4 Meshing

The scaffold and the patches were meshed using quadratic hexahedral 3D stress (C3D20) and piezoelectric elements (C3D20E), respectively, with a uniform element size of 0.1 mm. In addition, the patches were meshed with two elements over the thickness for any value selected for the patch thickness. The mesh convergence analysis was performed with respect to the developed electric potentials in the patches to identify the appropriate mesh size. The mesh was refined repeatedly until the difference between the highest electric potentials in two consecutive refinement steps was below 1%.

### 2.5 Parametric analysis

A parametric analysis was performed to investigate the effect of geometrical and material parameters on the generated voltage. These parameters included the pore’s radial size (*R*_1_ = 1 mm, *R*_2_ = 1.5 mm, and *R*_3_ = 2.5 mm), the pore’s vertical size (*V*_1_ = 1.7 mm, *V*_2_ = 3.1 mm), the number of pores in circumferential direction (*N*_1_ = 12, *N*_2_ = 8) and the piezoelectric patch thickness (*t*_1_ = 0.03 mm, *t*_2_ = 0.1 mm) (Figure 1A). In the parametric study, each of the investigated parameters is referred to as a factor, the values associated with each of them are called their levels, and each combination of a factor and levels is called an experiment. There are 24 *in silico* experiments in this study using different combinations of factors and levels as shown in Table 2. The highest voltage generated by the piezoelectric patches was determined for each experiment as an output variable and the effect of geometrical parameters for four different material groups (see Table 3) were investigated.

**TABLE 2:** Experiments generated with different combinations of geometrical factors and their levels.

Experiment	Pore’s radial size	Pore’s vertical size	Number of circumferential pores	Piezoelectric patch thickness
E1	*R*_1_	*V*_1_	*N*_1_	*t*_1_
E2	*R*_1_	*V*_1_	*N*_1_	*t*_2_
E3	*R*_1_	*V*_1_	*N*_2_	*t*_1_
E4	*R*_1_	*V*_1_	*N*_2_	*t*_2_
E5	*R*_1_	*V*_2_	*N*_1_	*t*_1_
E6	*R*_1_	*V*_2_	*N*_1_	*t*_2_
E7	*R*_1_	*V*_2_	*N*_2_	*t*_1_
E8	*R*_1_	*V*_2_	*N*_2_	*t*_2_
E9	*R*_2_	*V*_1_	*N*_1_	*t*_1_
E10	*R*_2_	*V*_1_	*N*_1_	*t*_2_
E11	*R*_2_	*V*_1_	*N*_2_	*t*_1_
E12	*R*_2_	*V*_1_	*N*_2_	*t*_2_
E13	*R*_2_	*V*_2_	*N*_1_	*t*_1_
E14	*R*_2_	*V*_2_	*N*_1_	*t*_2_
E15	*R*_2_	*V*_2_	*N*_2_	*t*_1_
E16	*R*_2_	*V*_2_	*N*_2_	*t*_2_
E17	*R*_3_	*V*_1_	*N*_1_	*t*_1_
E18	*R*_3_	*V*_1_	*N*_1_	*t*_2_
E19	*R*_3_	*V*_1_	*N*_2_	*t*_1_
E20	*R*_3_	*V*_1_	*N*_2_	*t*_2_
E21	*R*_3_	*V*_2_	*N*_1_	*t*_1_
E22	*R*_3_	*V*_2_	*N*_1_	*t*_2_
E23	*R*_3_	*V*_2_	*N*_2_	*t*_1_
E24	*R*_3_	*V*_2_	*N*_2_	*t*_2_

**TABLE 3:** Material groups.

	Scaffold material	Patches material
Group 1	Titanium	BT
Group 2	Titanium	PVDF
Group 3	PCL	BT
Group 4	PCL	PVDF

### 2.6 Data analysis

Analysis of variance (ANOVA) was used to investigate the significance and contribution of each factor for different material groups on the generated voltage. The total sum of squares of the deviation about the mean (*SS*_*T*_) was calculated as

$${SS}_{T}=\sum_{i=1}^{N}{\left(y_{i}-\bar{y}\right)}^{2}$$

Where *N* was the number of experiments, $y_{i}$ is the output (highest voltage value) for *i*th experiment, and $\bar{y}$ was the overall mean of highest voltage values. The sum of the squares of deviation about the mean for each factor (*SS*_*F*_) was

$${SS}_{F}=\sum_{i=1}^{n}{N_{F,i}\;\left({\bar{y}}_{F,i}-\bar{y}\right)}^{2}$$

where n is the number of levels for each factor, *N*_*F,i*_ is the number of experiments conducted for the $i^{th}$ level of each factor, i.e., 8 and 12 for the levels of factor *R* and factor *V*, respectively. ${\bar{y}}_{F,i}$ was calculated as the mean of output voltage for each level of each factor.

The percentage of the total sum of squares (*%TSS*) for each factor represented the contribution of each factor to the variance. It was considered as a measure of the importance of each factor calculated as (Borgiani et al., 2019)

$$\%TSS=\left({SS}_{F}/{SS}_{T}\right)100$$

## 3 Results

### 3.1 Mechanical behavior of different scaffolds designs

**FIGURE 2:**
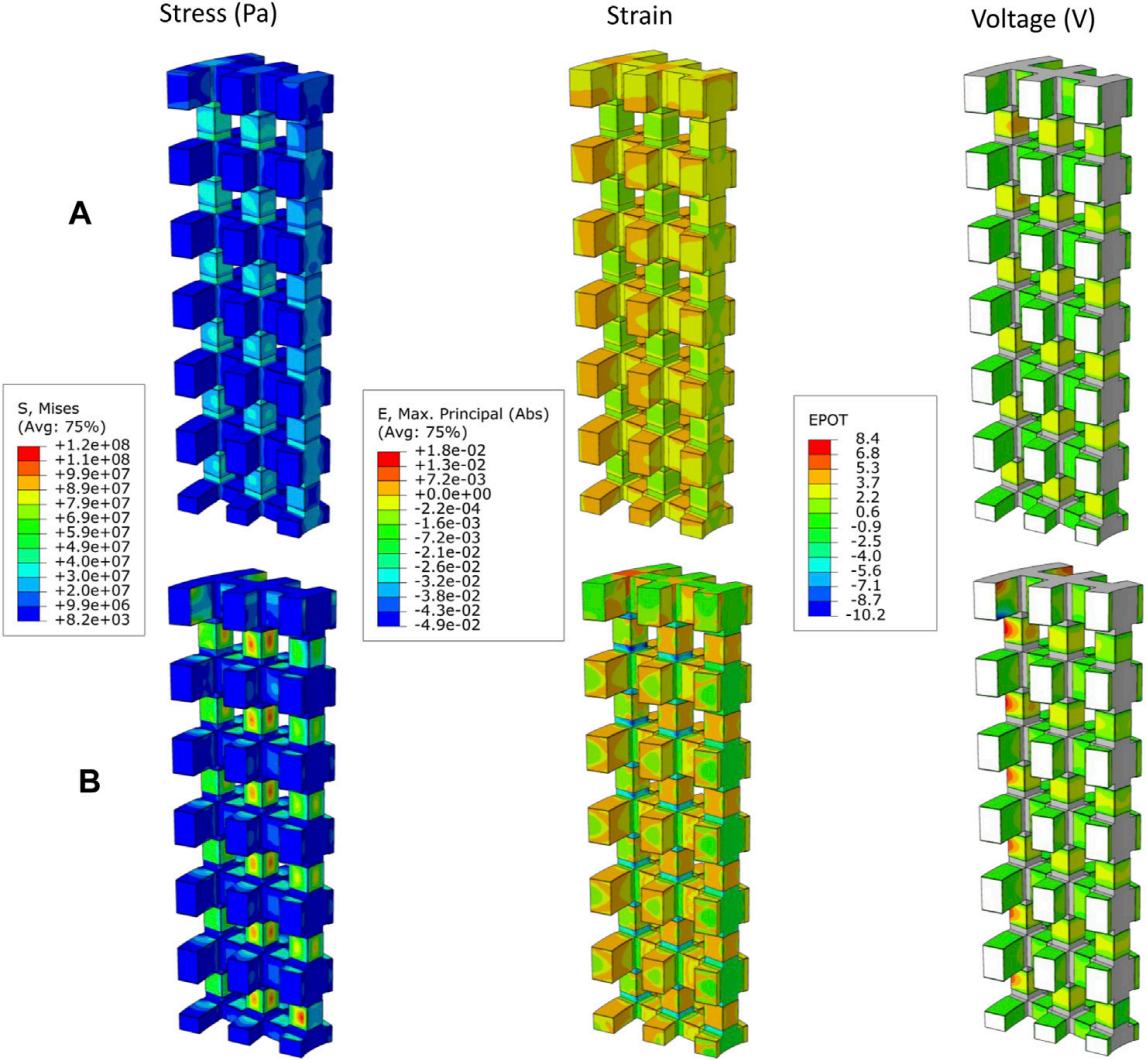
Distribution of von Mises stress (Pa), Absolute maximum principal strains, and generated voltage (V) by piezoelectric scaffold in case E9 where R = 1.5 mm, V = 1.7 mm, N = 12 and t = 0.03 mm with the **(A)** material group 1 (Titanium, BT), and **(B)** material group 3 (PCL, BT).

In Table 4, the apparent Young’s modulus of the scaffold for different material groups are shown for all experiments. The results show that in the material groups 1 and 2, the scaffolds had the highest apparent Young’s modulus, ranging from 18 to 31 GPa. In contrast, in material groups 3 and 4, the scaffolds resulted in a lower apparent Young modulus (0.06–0.17 GPa). Higher mechanical stresses were predicted in the vertical struts of the Ti scaffold (Figure 2A), while in the PCL scaffold, the patches were subjected to higher stresses as shown in Figure 2B. The horizontal struts of the scaffold experienced tensile strains while maximum compressive strains were predicted in the vertical struts.

**TABLE 4:** Apparent Young modulus of the piezoelectric scaffolds in all 24 experiments for different material groups.

Experiment	Scaffold apparent Young modulus (GPa)			
	Material group 1	Material group 2	Material group 3	Material group 4
E1	30	29	0.11	0.10
E2	31	29	0.12	0.10
E3	20	19	0.08	0.06
E4	21	19	0.08	0.07
E5	29	27	0.17	0.11
E6	30	27	0.17	0.11
E7	19	18	0.11	0.07
E8	20	18	0.11	0.08
E9	30	29	0.11	0.10
E10	31	29	0.12	0.10
E11	20	19	0.08	0.06
E12	21	19	0.08	0.07
E13	29	27	0.17	0.11
E14	30	27	0.17	0.11
E15	19	18	0.11	0.07
E16	20	18	0.11	0.08
E17	29	28	0.11	0.09
E18	31	28	0.11	0.10
E19	20	19	0.08	0.06
E20	21	19	0.08	0.07
E21	28	26	0.16	0.10
E22	30	26	0.17	0.11
E23	19	18	0.11	0.07
E24	20	18	0.11	0.07

### 3.2 Electrical behavior of different scaffolds designs

In the patches attached to the vertical struts positive voltage was produced while negative values were predicted in the patches located on the horizontal struts (Figure 2). The voltage generated by the piezoelectric patches was predicted for each of the experiments using the different material groups (Table 5). Higher values of voltage were predicted for scaffolds assigned with the material group 4 (PCL, PVDF) while the range of voltages generated by the first material group (Titanium, BT) were the lowest (Table 5).

**TABLE 5:** The voltage generated in the piezoelectric scaffold in different experiments using the defined material groups.

Experiment	Material group 1		Material group 2		Material group 3		Material group 4	
	Max negative voltage (V)	Max positive voltage (V)	Max negative voltage (V)	Max positive voltage (V)	Max negative voltage (V)	Max positive voltage (V)	Max negative voltage (V)	Max positive voltage (V)
E1	−2.2	4.2	−4.1	8.3	−10.8	8.6	−194	275
E2	−6.4	8.9	−13.9	26	−20.8	11.2	−385	453
E3	−5	6.2	−9.2	12.8	−24.8	22.1	−333	316
E4	−14.8	13.2	−31.4	40.3	−28.8	24.1	−690	534
E5	−2.1	4.2	−4	8.6	−10.1	13.5	−177	326
E6	−6.2	10.9	−13.5	26.5	−19.8	16.1	−351	641
E7	−4.9	6.2	−9	12.9	−21.9	20.2	−295	362
E8	−14.3	16.2	−30.6	39.5	−25.8	22.1	−607	729
E9	−2.2	4.2	−4	8.6	−10.2	8.3	−183	267
E10	−6.3	8.9	−13.7	27.3	−16.6	10.5	−364	437
E11	−5	6.4	−9.29	13.5	−21.3	18.8	−286	281
E12	−14.8	13.7	−31.5	42	−24.7	20.5	−593	469
E13	−2.1	4.4	−3.9	9.2	−8.9	12.3	−157	297
E14	−6.1	11	−13.3	28	−17.5	14.7	−311	586
E15	−4.9	6.5	−9	13.7	−19	17.4	−255	324
E16	−14.3	16.8	−30.7	41.6	−22.4	19.1	−526	650
E17	−2.1	4.5	−4	9.6	−8.3	7.2	−151	242
E18	−6.2	9.8	−13.5	30	−16.3	8.7	−300	375
E19	−5.2	7.1	−9.5	15	−19.6	17	−263	278
E20	−15	15	−32.2	46.7	−22.7	18.5	−545	446
E21	−2.1	4.8	−3.9	10.2	−7.6	11.2	−135	267
E22	−6	11.4	−13.2	30.9	−15.3	13.3	−268	532
E23	−4.9	7.1	−9	15.1	−14.5	13.4	−195	260
E24	−14.3	17.5	−30.7	45.6	−17.3	14.6	−402	520

**FIGURE 3:**
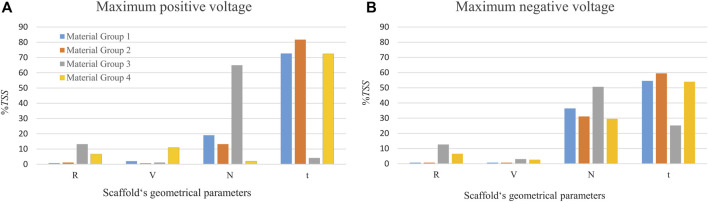
The percentage of the total sum of squares (%TSS) obtained from the ANOVA showing the effects of geometrical parameters on the **(A)** maximum positive voltage, and **(B)** maximum negative voltage produced in the scaffold. R and V are the pore’s radial size and the pore’s vertical size, respectively. N is the number of circumferential pores and t is the piezoelectric patch thickness. The material group 1 indicates combination of Ti and BT, the material group 2 contains Ti and PVDF, the material group 3 comprises PCL and BT, and the material group 4 is made of PCL and PVDF.

As shown in Figure 3, for material groups 1, 2 and 4, the thickness of the piezoelectric patches (*t*) was predicted to highly influence the amount of voltage produced in the scaffold (*%TSS ≥* 54). However, for the material group 3, the number of pores in the circumferential direction (*N*) showed the highest influence on the predicted voltage; followed by the radial size of the pores (*R*) for maximum positive voltage (Figure 3A) and the thickness of the patches (*t*) for the maximum negative voltage (Figure 3B). A small effect (*%TSS* ≤ 2.1) was determined by changing *R* (pore size in radial direction) and *V* (pore size in the vertical direction) in the material groups 1 and 2. In material group 3, *V* showed the lowest effect (*%TSS* ≤ 3.1). In material group 4, *N* and *V* showed the lowest effect for the maximum positive and negative voltages, respectively.

## 4 Discussion

The treatment of large bone defects still remains a clinical challenge. Considering the nature of piezoelectricity in biological tissues such as bone as well as its positive effect on bone regeneration, piezoelectric scaffolds appear as a promising alternative to current treatment strategies. In this study, we presented a novel scaffold design made of non-piezoelectric material, combined with piezoelectric patches attached to the surfaces facing the inner pores, which could produce voltages suitable for biological purposes. The influence of scaffold design parameters on the levels of electrical signals generated under physiological loading conditions was also investigated using computer models.

### 4.1 Mechanical behavior of the piezoelectric scaffold

To enhance large bone defect healing, scaffold structures must be primarily capable of providing mechanical stability and excellent mechanical stimulation without risking material failure. In this study, the mechanical analysis of the strut-like scaffolds revealed that the von Mises stress values of the scaffold, while subjected to the applied load, remained below the material’s yield strength (Figure 2). As shown in Table 4, the calculation of scaffolds apparent Young modulus showed that the scaffolds made of titanium are stiffer compared to the study of Pobloth et al. (2018) were the stiffness values of 0.82 and 2.88 GPa were reported for the two honeycomb titanium scaffolds implanted in the large bone defect in sheep simulated in that study. This can be attributed to the vertically oriented struts of the scaffold design in the current study which makes it structurally stiffer than honeycomb titanium scaffolds in vertical direction. However, as shown in Figure 2A, FE analyses predicted mechanical strains within the titanium scaffold pores of the present study (≤0.08%) comparable to those reported in their honeycomb titanium scaffold (≤0.15%) which was found to induce bone tissue regeneration (Pobloth et al., 2018). Computer model predictions of the mechanical strains in the PCL scaffold designed in this study were considerably higher (≤5%) for all configurations tested. In addition, PCL scaffolds apparent Young modulus falls within a similar range to the polyamide scaffolds reported in (Reznikov et al., 2019) which showed the highest bone ingrowth among the scaffolds with different stiffness levels. They reported an inverse correlation between stiffness and regenerative osteogenic response.

### 4.2 Electrical behavior of the piezoelectric scaffold

In our preliminary work (results not shown here), a strut-like scaffold design made of solely piezoelectric material was investigated to study its mechanical and electrical behavior. Finite element analyses showed that the mechanical behavior of the scaffold could be considered adequate for bone regeneration. However, predicted electrical signals (several kilovolts) were much higher than those reported in the literature to promote cellular activity. These results are in agreement with the findings of the work by Jiang et al. (2022).

In this study, computer model predictions showed that a strut-like scaffold made of non-piezoelectric material and piezoelectric patches attached to the surfaces facing the scaffold inner pores can result in voltages that range up to 729 V when titanium or PCL are used as scaffold material and BT or PVDF as piezoelectric material (Table 5). In the literature, surface electric potentials below 100 mV, which are in the range of natural endogenous potentials, have been reported to be suitable for osteogenic differentiation in composite piezoelectric membranes and films (Zhang et al., 2016; Genchi et al., 2018). Furthermore, direct electrical stimulation with higher voltages of up to 30 V have been used in *in vitro* studies leading to cell proliferation and viability (Dubey et al., 2011; Asri et al., 2022).

Depending on the poling direction, negative and positive charges are induced in the surface of piezoelectric materials under mechanical stress. Negatively charged surfaces have been demonstrated to favor cell proliferation in most experiments as reviewed in Baxter et al. (2010). However, the study of Vaněk et al. (2016) revealed that although both positive and negative charges are beneficial for cell attachment and subsequent growth, positive charges are slightly preferable since proteins mediating the cell adhesion are more easily adsorbed on positively charged surfaces due to the negative charge of these proteins. In this study, the patches attached to the vertical scaffold surfaces were subjected to compressive strains in the vertical direction and tensile strains in the perpendicular direction (in the direction of the thickness), inducing positive voltages. On the other hand, the patches attached to the horizontal struts experienced a reduction in thickness due to the in-plane tensile strains that caused negative voltages in these patches (Figure 2). However, the sign of generated voltages is interchangeable by reversing the poling direction in the patches.

### 4.3 Effect of material selection on the piezoelectric scaffold performance

In this study, we showed that the scaffold design made of the material group 1 (Titanium, BT) resulted in electrical signals ranges −15–17.5 V, which are within the range of those reported in the literature to stimulate cellular function (Dubey et al., 2011; Asri et al., 2022). Dubey et al. (2011) reported that cell proliferation was enhanced using electrical stimulations with a voltage range of 1–25 V. In addition, the adhesion and viability of human stem cells were further enhanced by applying a DC voltage of 30 V (Asri et al., 2022). The experimental values suitable for bone regeneration are summarized in Table 6. When using the material group 2 (Titanium, PVDF), we observed 2-to-3-fold increase in voltages compared with the material group 1. Although BT possesses higher piezoelectric constant (*d*_33_), its higher stiffness compared to PVDF results in lower strains and consequently lower voltages. In some *in vivo* studies, piezoelectric materials have been employed in the form of thin films (Zhang et al., 2016; Ribeiro et al., 2017) or as a coating on non-piezoelectric scaffolds (Liu et al., 2020a; Fan et al., 2020) in which a titanium alloy scaffold coated with BT have been shown to exhibit favorable osteogenic properties to enhance long-term bone formation.

**TABLE 6:** Electrical stimuli reported in the literature to be favorable for bone regeneration.

Study	Study description	Reported electrical stimuli
Genchi et al. (2018)	SaOS-2 osteoblast-like cell differentiation promoted by P(VDF-TrFE)/BNNT films in a culture test upon application of ultra sound stimulation	20–60 mV
Zhang et al. (2016)	Use of BTO NP/P(VDF-TrFE) nanocomposite membranes, where its surface potential was in the range of natural endogenous biopotential and encouraged fast and extensive bone defect healing *in vivo*	76 mV
Min et al. (2014)	The *in vitro* osteogenesis of the BMSCs or the MC3T3-E1 cells grown on the SPAN based interdigitated electrodes under the electrical stimulation revealed significantly increased mineralization by both cells compared to their respective controls	500 mV
Dubey et al. (2011)	A pulse electric field setup was used during cell culture experiments. The electrical stimulation was varied to study its influence on the proliferation of L929 mouse fibroblast cells on gelatin-coated control disc. L929 cells were then cultured on hydroxyapatite (HA) and HA-40 wt% BaTiO3 composite. Application of electric field during the cell culture experiment showed promotion of the cell proliferation and the cell spreading on the surface of the biomaterials	1–25 V
Asri et al. (2022)	Novel electroactive scaffolds were developed using the composites of Polycaprolactone (PCL) filled with conductive Thermally Reduced Graphene Oxide (TrGO) nanoparticles. Application of a DC voltage on the surface of the composite scaffolds further enhanced the adhesion and viability of human stem cells compared to pure PCL scaffolds with and without electrical stimulation	30 V

In this study, computer models of a scaffold made of the material group 3 (PCL, BT) predicted electrical signals in the range of those reported in the literature (Table 6), confirming suitability of BT as the piezoelectric material for patches in the current scaffold design. However, the scaffold made of the material group 4 (PCL, PVDF) provided voltages which did not fall within the range of those reported to be suitable for cellular activity. The predicted values considerably exceeded the desired range which can be attributed to the fact that Group 4, comprising the softest material combination in this study and therefore a high range of mechanical strain levels.

### 4.4 Sensitivity analysis

The parametric study performed in the current work has shown the thickness of the piezoelectric patches to be the most influential parameter on the amount of predicted voltage. On average, changing the patches thickness from 0.03 mm to 0.1 mm increased the voltage approximately 3 times, confirming that the amount of induced voltage under a constant mechanical stress is linearly proportional to the piezoelectric material thickness (Bernard et al., 2017). Regarding the other geometrical parameters, the number of pores in the circumferential direction (*N*) had a higher effect than the pore size. Reduction in *N* decreases the scaffold cross sectional area, which resist the compressive load, thus leading to less stiffness and higher strains, which in turn increased the voltages. This impact is higher in the scaffold made of PCL due to its low stiffness than Ti. Different combinations of scaffold design parameters in this study resulted in porosities ranging from 68% to 80%, which are in line with scaffolds that have shown a suitable regeneration potential (Liu et al., 2020b).

### 4.5 Limitations

In spite of the novel design of the piezoelectric scaffold reported in this work, this study holds some limitations as well. This study computationally demonstrated the potential of a piezoelectric scaffold design in generation of electrical signal for bone generation, but it has not been experimentally validated. Since the use of finite element modelling techniques for the quantification of electrical and mechanical signals in different structures has been widely validated, we can therefore assume that the values predicted by the computer model are close to reality. The manufacturing of the scaffold was also not evaluated in this study. Although manufacturing scaffolds with patches attached to their surfaces might appear challenging, there are already several approaches that have been successfully implemented in the literature. As one possible solution, the scaffold structure could be assembled with 3D printed LEGO-like smaller pieces of the scaffold containing the patches (Hipfinger et al., 2020). Alternatively, fabrication of these scaffolds can be performed using the technique presented in Liu et al. (2020b) where they additively manufactured a Ti6Al4V-based scaffold which was then coated with barium titanate using hydrothermal synthesis. They then applied a corona poling to change the polarization of the piezoelectric coating. Another limitation is that the scaffold was designed to fill a large bone defect in a large animal model. The translation of these results to patients remains open. However, the use of a pre-clinical animal study allows a better comparison to the experimental literature.

The potential of the piezoelectric scaffolds have been experimentally investigated for tissue regeneration including bone, nerves, etc. (Tandon et al., 2018). However, the induced electromechanical signals within these scaffolds remain largely unknown due to the difficulties in the measurement of these signals. Therefore, the computational modelling of piezoelectric scaffolds can certainly provide valuable insights, while reducing the time-consuming and ethically-problematic experimental efforts. This model has a high potential to be used to simulate small and large bone defects in small or large animal models.

## 5 Concluding remarks

Piezoelectric materials, as a class of smart materials, have attracted significant interest in the field of tissue engineering owing to their capability to support regeneration processes. Although it has been shown that electrical signals influence cellular behavior, the effect of scaffold design on the generated electrical and mechanical signals remains poorly understood.

In the current preclinical study, we investigated the mechanical and electrical signals induced within a novel piezoelectric scaffold design, using computer modelling approaches. Our findings showed:

• A strut-like scaffold made of titanium or PCL with BT piezoelectric patches results in electromechanical signals within the range of those reported to promote cellular activity.
• The thickness of the piezoelectric patches in a novel strut-like scaffold plays a key role on the generated electrical signals.

This study provides insights into the design of piezoelectric scaffolds and the influence of design parameters on the generated electrical and mechanical signals. Future studies should focus on understanding the ranges of electrical signals promoting bone regeneration and the optimization of the scaffold designs to achieve enhanced bone regeneration.

## Data Availability

The original contributions presented in the study are included in the article/Supplementary Material, further inquiries can be directed to the corresponding author.

## References

[B1] Akkouch A. Zhang Z. Rouabhia M. (2011). A novel collagen/hydroxyapatite/poly (lactide‐co‐ε‐caprolactone) biodegradable and bioactive 3D porous scaffold for bone regeneration. J. Biomed. Mater. Res. Part A 96, 693–704. 10.1002/jbm.a.33033 21284080

[B2] Arjunan A. Demetriou M. Baroutaji A. Wang C. (2020). Mechanical performance of highly permeable laser melted Ti6Al4V bone scaffolds. J. Mech. Behav. Biomed. Mater. 102, 103517. 10.1016/j.jmbbm.2019.103517 31877520

[B3] Asri N. A. N. Mahat M. M. Zakaria A. Safian M. F. Abd Hamid U. M. (2022). Fabrication methods of electroactive scaffold-based conducting polymers for tissue engineering application: a review. Front. Bioeng. Biotechnol. 10, 876696. 10.3389/fbioe.2022.876696 35875482 PMC9300926

[B4] Azimi B. Milazzo M. Lazzeri A. Berrettini S. Uddin M. J. Qin Z. (2020). Electrospinning piezoelectric fibers for biocompatible devices. Adv. Healthc. Mater. 9, 1901287. 10.1002/adhm.201901287 PMC6949425 31701671

[B5] Baxter F. R. Bowen C. R. Turner I. G. Dent A. C. E. (2010). Electrically active bioceramics: a review of interfacial responses. Ann. Biomed. Eng. 38, 2079–2092. 10.1007/s10439-010-9977-6 20198510

[B6] Bernard F. Gimeno L. Viala B. Gusarov B. Cugat O. (2017). Direct piezoelectric coefficient measurements of PVDF and PLLA under controlled strain and stress. Multidiscip. Digit. Publ. Inst. Proc. 1, 335. 10.3390/proceedings1040335

[B7] Borgiani E. Figge C. Kruck B. Willie B. M. Duda G. N. Checa S. (2019). Age‐related changes in the mechanical regulation of bone healing are explained by altered cellular mechanoresponse. J. Bone Mineral Res. 34, 1923–1937. 10.1002/jbmr.3801 31121071

[B8] Bounds E. J. Khan M. Kok S. J. (2018). Electrical burns, StatPearls. 30137799

[B9] Breuls R. G. M. Jiya T. U. Smit T. H. (2008). Scaffold stiffness influences cell behavior: opportunities for skeletal tissue engineering. open Orthop. J. 2, 103–109. 10.2174/1874325000802010103 19478934 PMC2687114

[B10] BußMANN A. Leistner H. Zhou D. Wackerle M. Congar Y. Richter M. (2021). Piezoelectric silicon micropump for drug delivery applications. Appl. Sci. 11, 8008. 10.3390/app11178008

[B11] Carlier A. Geris L. Lammens J. Van Oosterwyck H. (2015). Bringing computational models of bone regeneration to the clinic. Wiley Interdiscip. Rev. Syst. Biol. Med. 7, 183–194. 10.1002/wsbm.1299 25903383

[B12] Cerrolaza M. Duarte V. Garzón-Alvarado D. (2017). Analysis of bone remodeling under piezoelectricity effects using boundary elements. J. Bionic Eng. 14, 659–671. 10.1016/s1672-6529(16)60432-8

[B13] Chen H. Han Q. Wang C. Liu Y. Chen B. Wang J. (2020). Porous scaffold design for additive manufacturing in orthopedics: a review. Front. Bioeng. Biotechnol. 8, 609. 10.3389/fbioe.2020.00609 32626698 PMC7311579

[B14] Ciombor D. M. Aaron R. K. (2005). The role of electrical stimulation in bone repair. Foot ankle Clin. 10, 579–593. 10.1016/j.fcl.2005.06.006 16297820

[B15] D’Alessandro D. Ricci C. Milazzo M. Strangis G. Forli F. Buda G. (2021). Piezoelectric signals in vascularized bone regeneration. Biomolecules 11, 1731. 10.3390/biom11111731 34827729 PMC8615512

[B16] Damaraju S. M. Shen Y. Elele E. Khusid B. Eshghinejad A. Li J. (2017). Three-dimensional piezoelectric fibrous scaffolds selectively promote mesenchymal stem cell differentiation. Biomaterials 149, 51–62. 10.1016/j.biomaterials.2017.09.024 28992510

[B17] Deng F. Liu L. Li Z. Liu J. (2021). 3D printed Ti6Al4V bone scaffolds with different pore structure effects on bone ingrowth. J. Biol. Eng. 15, 4–13. 10.1186/s13036-021-00255-8 33478505 PMC7818551

[B18] Dubey A. K. Gupta S. D. Basu B. (2011). Optimization of electrical stimulation parameters for enhanced cell proliferation on biomaterial surfaces. J. Biomed. Mater. Res. Part B Appl. Biomaterials 98, 18–29. 10.1002/jbm.b.31827 21432997

[B19] Eshraghi S. Das S. (2010). Mechanical and microstructural properties of polycaprolactone scaffolds with one-dimensional, two-dimensional, and three-dimensional orthogonally oriented porous architectures produced by selective laser sintering. Acta biomater. 6, 2467–2476. 10.1016/j.actbio.2010.02.002 20144914 PMC2874084

[B20] Fan B. Guo Z. Li X. Li S. Gao P. Xiao X. (2020). Electroactive barium titanate coated titanium scaffold improves osteogenesis and osseointegration with low-intensity pulsed ultrasound for large segmental bone defects. Bioact. Mater. 5, 1087–1101. 10.1016/j.bioactmat.2020.07.001 32695938 PMC7363989

[B21] FERNÁNDEZ J. R. García-Aznar J. M. Martínez R. (2012). Piezoelectricity could predict sites of formation/resorption in bone remodelling and modelling. J. Theor. Biol. 292, 86–92. 10.1016/j.jtbi.2011.09.032 22001080

[B22] García-Gareta E. Coathup M. J. Blunn G. W. (2015). Osteoinduction of bone grafting materials for bone repair and regeneration. Bone 81, 112–121. 10.1016/j.bone.2015.07.007 26163110

[B23] Garzón-Alvarado D. A. Ramírez-Martínez A. M. Cardozo De Martínez C. A. (2012). Numerical test concerning bone mass apposition under electrical and mechanical stimulus. Theor. Biol. Med. Model. 9, 14–17. 10.1186/1742-4682-9-14 22578031 PMC3502529

[B24] Genchi G. G. Sinibaldi E. Ceseracciu L. Labardi M. Marino A. Marras S. (2018). Ultrasound-activated piezoelectric P (VDF-TrFE)/boron nitride nanotube composite films promote differentiation of human SaOS-2 osteoblast-like cells. Nanomedicine Nanotechnol. Biol. Med. 14, 2421–2432. 10.1016/j.nano.2017.05.006 28552646

[B25] Geris L. Reed A. A. C. Vander Sloten J. Simpson A. H. R. Van Oosterwyck H. (2010). Occurrence and treatment of bone atrophic non-unions investigated by an integrative approach. PLoS Comput. Biol. 6, e1000915. 10.1371/journal.pcbi.1000915 20824125 PMC2932678

[B26] Goonoo N. Bhaw-Luximon A. (2022). Piezoelectric polymeric scaffold materials as biomechanical cellular stimuli to enhance tissue regeneration. Mater. Today Commun. 31, 103491. 10.1016/j.mtcomm.2022.103491

[B27] Hipfinger C. Subbiah R. Tahayeri A. Athirasala A. Horsophonphong S. Thrivikraman G. (2020). 3D printing of microgel-loaded modular LEGO-like cages as instructive scaffolds for tissue engineering. bioRxiv, 1–19. 10.1101/2020.03.02.974204 32700332

[B28] Hu W. W. Chen T. C. Tsao C. W. Cheng Y. C. (2019). The effects of substrate‐mediated electrical stimulation on the promotion of osteogenic differentiation and its optimization. J. Biomed. Mater. Res. Part B Appl. Biomaterials 107, 1607–1619. 10.1002/jbm.b.34253 30318825

[B29] Huang B. Vyas C. Roberts I. Poutrel Q.-A. Chiang W.-H. Blaker J. J. (2019). Fabrication and characterisation of 3D printed MWCNT composite porous scaffolds for bone regeneration. Mater. Sci. Eng. C 98, 266–278. 10.1016/j.msec.2018.12.100 30813027

[B30] Jaber M. Poh P. S. Duda G. N. Checa S. (2022). PCL strut-like scaffolds appear superior to gyroid in terms of bone regeneration within a long bone large defect: an *in silico* study. Front. Bioeng. Biotechnol. 10, 995266. 10.3389/fbioe.2022.995266 36213070 PMC9540363

[B31] Jiang Z. Cheng L. Zeng Y. Zhang Z. Zhao Y. Dong P. (2022). 3D printing of porous scaffolds BaTiO3 piezoelectric ceramics and regulation of their mechanical and electrical properties. Ceram. Int. 48, 6477–6487. 10.1016/j.ceramint.2021.11.192

[B32] Khalifeh J. M. Zohny Z. Macewan M. Stephen M. Johnston W. Gamble P. (2018). Electrical stimulation and bone healing: a review of current technology and clinical applications. IEEE Rev. Biomed. Eng. 11, 217–232. 10.1109/rbme.2018.2799189 29994564

[B33] Lay R. Deijs G. S. Malmström J. (2021). The intrinsic piezoelectric properties of materials–a review with a focus on biological materials. RSC Adv. 11, 30657–30673. 10.1039/d1ra03557f 35498945 PMC9041315

[B34] Leppik L. Zhihua H. Mobini S. Thottakkattumana Parameswaran V. Eischen-Loges M. Slavici A. (2018). Combining electrical stimulation and tissue engineering to treat large bone defects in a rat model. Sci. Rep. 8, 6307–6314. 10.1038/s41598-018-24892-0 29679025 PMC5910383

[B35] Li J. Liu X. Crook J. M. Wallace G. G. (2020). Electrical stimulation-induced osteogenesis of human adipose derived stem cells using a conductive graphene-cellulose scaffold. Mater. Sci. Eng. C 107, 110312. 10.1016/j.msec.2019.110312 31761174

[B36] Liu H. Peng H. Wu Y. Zhang C. Cai Y. Xu G. (2013). The promotion of bone regeneration by nanofibrous hydroxyapatite/chitosan scaffolds by effects on integrin-BMP/Smad signaling pathway in BMSCs. Biomaterials 34, 4404–4417. 10.1016/j.biomaterials.2013.02.048 23515177

[B37] Liu H. Zhong J. Lee C. Lee S.-W. Lin L. (2018). A comprehensive review on piezoelectric energy harvesting technology: materials, mechanisms, and applications. Appl. Phys. Rev. 5, 041306. 10.1063/1.5074184

[B38] Liu W. Li X. Jiao Y. Wu C. Guo S. Xiao X. (2020a). Biological effects of a three-dimensionally printed Ti6Al4V scaffold coated with piezoelectric BaTiO3 nanoparticles on bone formation. ACS Appl. Mater. Interfaces 12, 51885–51903. 10.1021/acsami.0c10957 33166458

[B39] Liu W. Yang D. Wei X. Guo S. Wang N. Tang Z. (2020b). Fabrication of piezoelectric porous BaTiO3 scaffold to repair large segmental bone defect in sheep. J. Biomaterials Appl. 35, 544–552. 10.1177/0885328220942906 32660363

[B40] Marques-Almeida T. Cardoso V. F. Gama M. Lanceros-Mendez S. Ribeiro C. (2020). Patterned piezoelectric scaffolds for osteogenic differentiation. Int. J. Mol. Sci. 21, 8352. 10.3390/ijms21218352 33171761 PMC7672637

[B41] Metz C. Duda G. N. Checa S. (2020). Towards multi-dynamic mechano-biological optimization of 3D-printed scaffolds to foster bone regeneration. Acta biomater. 101, 117–127. 10.1016/j.actbio.2019.10.029 31669697

[B42] Milovanović J. Stojković M. Trifunović M. Vitković N. (2020). Review of bone scaffold design concepts and design methods. Facta Univ. Ser. Mech. Eng. 151-173. 10.22190/FUME200328038M

[B43] Min Y. Liu Y. Poojari Y. Wu J.-C. Hildreth B. E. III Rosol T. J. (2014). Self-doped polyaniline-based interdigitated electrodes for electrical stimulation of osteoblast cell lines. Synth. Met. 198, 308–313. 10.1016/j.synthmet.2014.10.035

[B44] More N. Srivastava A. Kapusetti G. (2020). Graphene oxide reinforcement enhances the piezoelectric and mechanical properties of poly (3-hydroxybutyrate-co-3-hydroxy valerate)-based nanofibrous scaffolds for improved proliferation of chondrocytes and ECM production. ACS Appl. Bio Mater. 3, 6823–6835. 10.1021/acsabm.0c00765 35019345

[B45] Narita F. Shindo Y. (2015). Piezoelectric detection and response characteristics of barium titanate unimorph cantilevers under AC electric fields. Int. J. Metall. Mat. Eng. 1, 103. 10.15344/2455-2372/2015/103

[B46] Nicholson J. A. Makaram N. Simpson A. Keating J. F. (2021). Fracture nonunion in long bones: a literature review of risk factors and surgical management. Injury 52, S3–S11. 10.1016/j.injury.2020.11.029 33221036

[B47] Nix E. L. Ward I. (1986). The measurement of the shear piezoelectric coefficients of polyvinylidene fluoride. Ferroelectrics 67, 137–141. 10.1080/00150198608245016

[B48] Noll M.-U. Lentz L. Wagner U. V. (2019). On the discretization of a bistable cantilever beam with application to energy harvesting. Facta Univ. Ser. Mech. Eng. 17, 125–139. 10.22190/fume190301031n

[B49] Park J. B. Kelly B. J. Kenner G. H. Recum A. V. Grether M. F. Coffeen W. W. (1981). Piezoelectric ceramic implants: *in vivo* results. J. Biomed. Mater. Res. 15, 103–110. 10.1002/jbm.820150114 7348700

[B50] Perier-Metz C. Cipitria A. Hutmacher D. W. Duda G. N. Checa S. (2022). An *in silico* model predicts the impact of scaffold design in large bone defect regeneration. Acta Biomater. 145, 329–341. 10.1016/j.actbio.2022.04.008 35417799

[B51] Perier-Metz C. Duda G. N. Checa S. (2020). Mechano-biological computer model of scaffold-supported bone regeneration: effect of bone graft and scaffold structure on large bone defect tissue patterning. Front. Bioeng. Biotechnol. 1245, 585799. 10.3389/fbioe.2020.585799 PMC7686036 33262976

[B52] Pobloth A.-M. Checa S. Razi H. Petersen A. Weaver J. C. Schmidt-Bleek K. (2018). Mechanobiologically optimized 3D titanium-mesh scaffolds enhance bone regeneration in critical segmental defects in sheep. Sci. Transl. Med. 10, eaam8828. 10.1126/scitranslmed.aam8828 29321260

[B53] Podshivalov L. Fischer A. Bar-Yoseph P. Z. (2014). On the road to personalized medicine: multiscale computational modeling of bone tissue. Archives Comput. Methods Eng. 21, 399–479. 10.1007/s11831-014-9120-1

[B54] Polley C. Distler T. Detsch R. Lund H. Springer A. Boccaccini A. R. (2020). 3D printing of piezoelectric barium titanate-hydroxyapatite scaffolds with interconnected porosity for bone tissue engineering. Materials 13, 1773. 10.3390/ma13071773 32283869 PMC7179021

[B55] Qi F. Zeng Z. Yao J. Cai W. Zhao Z. Peng S. (2021). Constructing core-shell structured BaTiO3@ carbon boosts piezoelectric activity and cell response of polymer scaffolds. Mater. Sci. Eng. C 126, 112129. 10.1016/j.msec.2021.112129 34082946

[B56] Qing X. Li W. Wang Y. Sun H. (2019). Piezoelectric transducer-based structural health monitoring for aircraft applications. Sensors 19, 545. 10.3390/s19030545 30696061 PMC6387086

[B57] Qu C. Qin Q.-H. Kang Y. (2006). A hypothetical mechanism of bone remodeling and modeling under electromagnetic loads. Biomaterials 27, 4050–4057. 10.1016/j.biomaterials.2006.03.015 16574223

[B58] Rajabi A. H. Jaffe M. Arinzeh T. L. (2015). Piezoelectric materials for tissue regeneration: a review. Acta biomater. 24, 12–23. 10.1016/j.actbio.2015.07.010 26162587

[B59] Razi H. Checa S. Schaser K. D. Duda G. N. (2012). Shaping scaffold structures in rapid manufacturing implants: a modeling approach toward mechano‐biologically optimized configurations for large bone defect. J. Biomed. Mater. Res. Part B Appl. Biomaterials 100, 1736–1745. 10.1002/jbm.b.32740 22807248

[B60] Reznikov N. Boughton O. R. Ghouse S. Weston A. E. Collinson L. Blunn G. W. (2019). Individual response variations in scaffold-guided bone regeneration are determined by independent strain-and injury-induced mechanisms. Biomaterials 194, 183–194. 10.1016/j.biomaterials.2018.11.026 30611115 PMC6345626

[B61] Ribeiro C. Correia D. M. Rodrigues I. Guardão L. Guimarães S. Soares R. (2017). *In vivo* demonstration of the suitability of piezoelectric stimuli for bone reparation. Mater. Lett. 209, 118–121. 10.1016/j.matlet.2017.07.099

[B62] Sanz-Sánchez I. Sanz-Martín I. Ortiz-Vigón A. Molina A. Sanz M. (2022). Complications in bone‐grafting procedures: classification and management. Periodontology 88, 86–102. 10.1111/prd.12413 35103322

[B63] Shick T. M. Abdul Kadir A. Z. Ngadiman N. H. A. Ma’Aram A. (2019). A review of biomaterials scaffold fabrication in additive manufacturing for tissue engineering. J. Bioact. Compatible Polym. 34, 415–435. 10.1177/0883911519877426

[B64] Stapleton A. Noor M. R. Soulimane T. Tofail S. A. (2016). Physiological role of piezoelectricity in biological building blocks. Electrically active materials for medical devices. World Scientific.

[B65] Tandon B. Blaker J. J. Cartmell S. H. (2018). Piezoelectric materials as stimulatory biomedical materials and scaffolds for bone repair. Acta biomater. 73, 1–20. 10.1016/j.actbio.2018.04.026 29673838

[B66] Tan X. Obaid R. F. Smaisim G. F. Esfahani M. M. Alsaikhan F. Baghaei S. (2023). Investigation of addition of calcium phosphate ceramic to multilayer scaffold for bone applications with improved mechanical properties: fuzzy logic analysis. Ceram. Int. 49, 8339–8349. 10.1016/j.ceramint.2022.10.366

[B67] Tariverdian T. Behnamghader A. Milan P. B. Barzegar-Bafrooei H. Mozafari M. (2019). 3D-printed barium strontium titanate-based piezoelectric scaffolds for bone tissue engineering. Ceram. Int. 45, 14029–14038. 10.1016/j.ceramint.2019.04.102

[B68] Topolov V. Y. Bowen C. R. Bisegna P. (2015). New aspect-ratio effect in three-component composites for piezoelectric sensor, hydrophone and energy-harvesting applications. Sensors Actuators A Phys. 229, 94–103. 10.1016/j.sna.2015.03.025

[B69] Vaněk P. Kolská Z. Luxbacher T. García J. A. L. Lehocký M. Vandrovcová M. (2016). Electrical activity of ferroelectric biomaterials and its effects on the adhesion, growth and enzymatic activity of human osteoblast-like cells. J. Phys. D Appl. Phys. 49, 175403. 10.1088/0022-3727/49/17/175403

[B70] Wildemann B. Ignatius A. Leung F. Taitsman L. A. Smith R. M. Pesántez R. (2021). Non-union bone fractures. Nat. Rev. Dis. Prim. 7, 57–21. 10.1038/s41572-021-00289-8 34354083

[B71] Zadpoor A. A. (2015). Bone tissue regeneration: the role of scaffold geometry. Biomaterials Sci. 3, 231–245. 10.1039/c4bm00291a 26218114

[B72] Zaszczynska A. Sajkiewicz P. Gradys A. (2020). Piezoelectric scaffolds as smart materials for neural tissue engineering. Polymers 12, 161. 10.3390/polym12010161 31936240 PMC7022784

[B73] Zhang X. Zhang C. Lin Y. Hu P. Shen Y. Wang K. (2016). Nanocomposite membranes enhance bone regeneration through restoring physiological electric microenvironment. ACS Nano 10, 7279–7286. 10.1021/acsnano.6b02247 27389708

[B74] Zhang Y. Chen L. Zeng J. Zhou K. Zhang D. (2014). Aligned porous barium titanate/hydroxyapatite composites with high piezoelectric coefficients for bone tissue engineering. Mater. Sci. Eng. C 39, 143–149. 10.1016/j.msec.2014.02.022 24863210

[B75] Zhao F. Zhang C. Liu J. Liu L. Cao X. Chen X. (2020). Periosteum structure/function-mimicking bioactive scaffolds with piezoelectric/chem/nano signals for critical-sized bone regeneration. Chem. Eng. J. 402, 126203. 10.1016/j.cej.2020.126203

[B76] Zimmermann G. Moghaddam A. (2011). Allograft bone matrix versus synthetic bone graft substitutes. Injury 42, 16–21. 10.1016/j.injury.2011.06.199 21889142

