# Supplementary material for: Computational design and evaluation of the mechanical and electrical behavior of a piezoelectric scaffold: a preclinical study
Source: Front Bioeng Biotechnol. 2024 Jan 11;11:1261108. doi: 10.3389/fbioe.2023.1261108 (PMC10808828; doi:10.3389/fbioe.2023.1261108)
Supplement: Supplementary file 1 [file DataSheet1.PDF]

Supplementary Material

The finite element model of the large bone defect of previous study (Perier-Metz et al., 2020) was used to determine the load shares of the scaffold and fixation plate. The scaffold design proposed in the current study was replaced in that model to calculate the load shares of the scaffold and fixation plate under applied physiological loadings (compression and the bending applied on the top surface of the bone) using definition of output sections for both components as shown in **Supplementary Figure 1.**

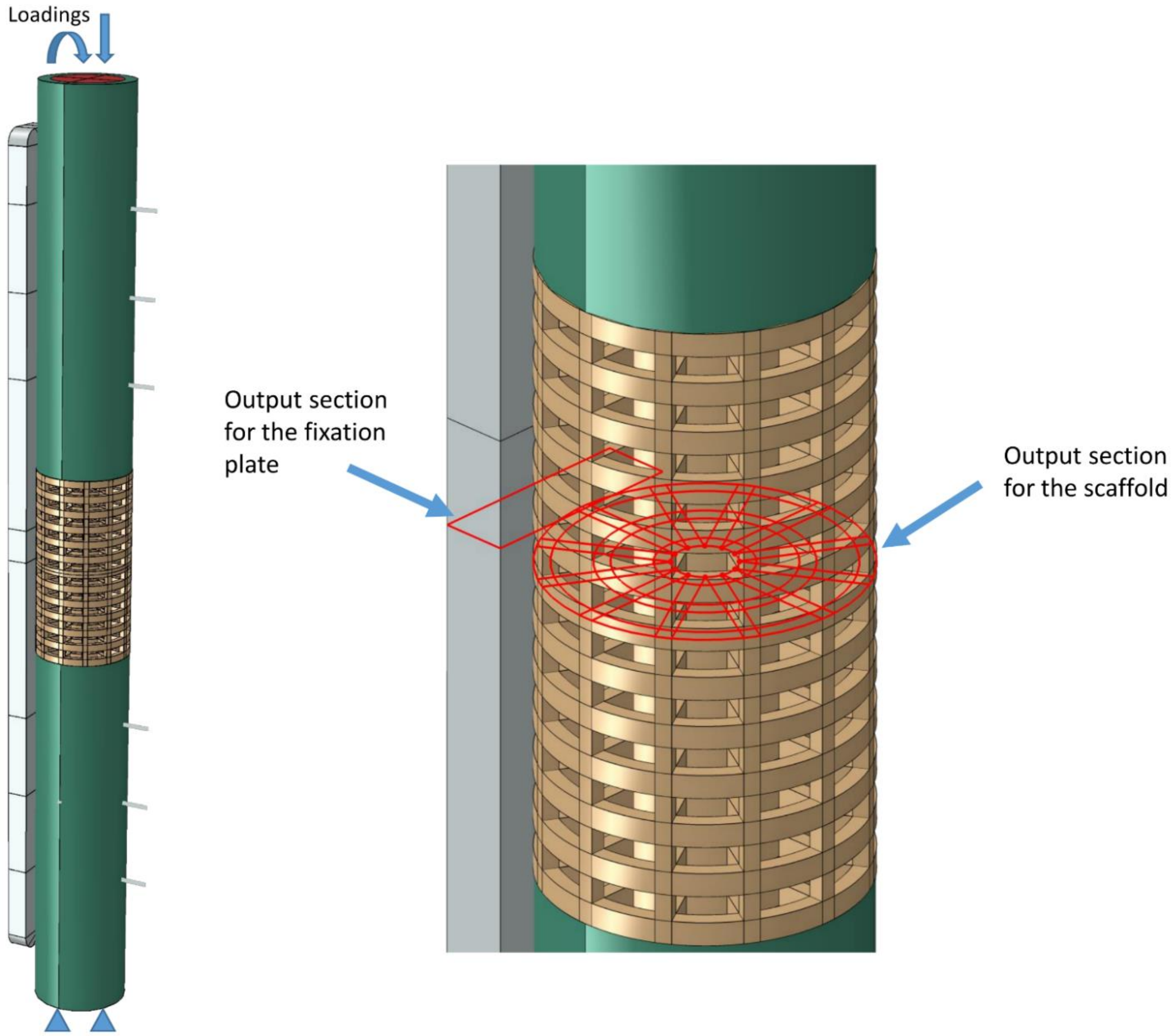

Supplementary Figure 1. Determination of load shares of the scaffold and fixation plate in the finite element model of the large bone defect under applied physiological loadings using definition of output sections for both components.

Supplementary Table 1. The load shares of the scaffold and fixation plate determined for the different models of scaffold design configurations.

Model NO.	Pore's radial size	Pore's vertical size	Number of circumferential pores	Scaffold's Material	Shared Load born by the Scaffold (N)	Shared Load born by the Fixation Plate (N)
1	R_1	V_1	N_1	Ti	1100	275
2	R_1	V_1	N_1	PCL	240	1135
3	R_1	V_1	N_2	Ti	1095	280
4	R_1	V_1	N_2	PCL	180	1195
5	R_1	V_2	N_1	Ti	1098	277
6	R_1	V_2	N_1	PCL	230	1145
7	R_1	V_2	N_2	Ti	1090	285
8	R_1	V_2	N_2	PCL	165	1210
9	R_2	V_1	N_1	Ti	1097	278
0	R_2	V_1	N_1	PCL	225	1150
11	R_2	V_1	N_2	Ti	1088	287
12	R_2	V_1	N_2	PCL	155	1220
13	R_2	V_2	N_1	Ti	1092	283
14	R_2	V_2	N_1	PCL	205	1170
15	R_2	V_2	N_2	Ti	1082	293
16	R_2	V_2	N_2	PCL	145	1230
17	R_3	V_1	N_1	Ti	1094	281
18	R_3	V_1	N_1	PCL	190	1185
19	R_3	V_1	N_2	Ti	1084	291
20	R_3	V_1	N_2	PCL	140	1235
21	R_3	V_2	N_1	Ti	1088	287
22	R_3	V_2	N_1	PCL	180	1195
23	R_3	V_2	N_2	Ti	1058	317
24	R_3	V_2	N_2	PCL	110	1265
